# Learning to Avoid Obstacles With Minimal Intervention Control

**DOI:** 10.3389/frobt.2020.00060

**Published:** 2020-05-28

**Authors:** Anqing Duan, Raffaello Camoriano, Diego Ferigo, Yanlong Huang, Daniele Calandriello, Lorenzo Rosasco, Daniele Pucci

**Affiliations:** ^1^Dynamic Interaction Control Lab, Italian Institute of Technology, Genoa, Italy; ^2^DIBRIS, Università degli Studi di Genova, Genoa, Italy; ^3^Laboratory for Computational and Statistical Learning (IIT@MIT), Istituto Italiano di Tecnologia and Massachusetts Institute of Technology, Cambridge, MA, United States; ^4^Machine Learning and Optimization Group, School of Computer Science, University of Manchester, Manchester, United Kingdom; ^5^Department of Advanced Robotics, Istituto Italiano di Tecnologia, Genova, Italy; ^6^Machine Learning Genoa (MaLGa) Center, Università di Genova, Genoa, Italy

**Keywords:** programming by demonstration, reinforcement learning, obstacle avoidance, humanoid robots, minimal intervention control

## Abstract

Programming by demonstration has received much attention as it offers a general framework which allows robots to efficiently acquire novel motor skills from a human teacher. While traditional imitation learning that only focuses on either Cartesian or joint space might become inappropriate in situations where both spaces are equally important (e.g., writing or striking task), hybrid imitation learning of skills in both Cartesian and joint spaces simultaneously has been studied recently. However, an important issue which often arises in dynamical or unstructured environments is overlooked, namely how can a robot avoid obstacles? In this paper, we aim to address the problem of avoiding obstacles in the context of hybrid imitation learning. Specifically, we propose to tackle three subproblems: (i) designing a proper potential field so as to bypass obstacles, (ii) guaranteeing joint limits are respected when adjusting trajectories in the process of avoiding obstacles, and (iii) determining proper control commands for robots such that potential human-robot interaction is safe. By solving the aforementioned subproblems, the robot is capable of generalizing observed skills to new situations featuring obstacles in a feasible and safe manner. The effectiveness of the proposed method is validated through a toy example as well as a real transportation experiment on the iCub humanoid robot.

## 1. Introduction

Over the past few years, there has been growing demand for bringing robots from industrial manufacturing lines to human-centered scenarios thanks to ever evolving sensors, actuators, and processors. Increasing computational power also gives rise to novel control and learning algorithms. Nevertheless, contrary to the high maturity of industrial robots and relatively simple service robots, how to deploy general purpose robots, such as humanoid robots into cluttered environments still remains a formidable challenge. Conceivably, before humanoids can successfully operate in daily-life settings, a series of challenges need to be confronted. One of the major concerns for robots to operate outside laboratory environments is obstacle avoidance. Indeed, obstacle avoidance represents a necessary capability for robots to become more autonomous, flexible and safer in order to cope with complex working environments. In addition, as humanoid robots could sometimes be expected to work alongside human beings, classical high-gain feedback control shall be deprecated in case human-robot interaction becomes dangerous. Hence, compliance is a highly desired skill in this case, as the incorporation of variable impedance skills into the robot controller allows for a safe physical human-robot interaction (Abu-Dakka et al., 2018).

In view of high-dimensional state and action spaces of humanoid robots, a user-friendly method to endow them with various skills is Programming by Demonstration (PbD), also known as imitation learning (Billard et al., 2008). The complete procedure of PbD typically consists of a demonstration phase, where robots are shown the desired behavior, and a reproduction phase, where robots are required to reproduce the learned skills, typically with the help of movement primitives (Ijspeert et al., 2013; Huang et al., 2019). Under the framework of PbD, humanoid robots are able to efficiently acquire novel motor skills from demonstrations of a human teacher. Following this paradigm, many successful results have been achieved, such as peg-in-hole task (Abu-Dakka et al., 2014), cleaning task (Duan et al., 2019), table tennis (Huang et al., 2016), etc.

Traditional imitation learning that only focuses on either Cartesian or joint space might become inappropriate in situations where both spaces are equally important, such as in writing or striking tasks. Consequently, hybrid imitation learning of skills in both Cartesian and joint spaces simultaneously has recently emerged. In order to further generalize the applicability of hybrid imitation learning, this paper considers the integration of obstacle avoidance in the context of hybrid imitation learning such that robots can reproduce the learned skills in a broader range of situations. Specifically, we consider the following three aspects: (i) designing a proper potential field so as to bypass obstacles. As a common technique for obstacle avoidance within PbD, the potential field formulation as well as its hyperparameters play an important role in realizing obstacle avoidance. In order to compare the performance of different potential fields, a novel imitation metric is proposed. Moreover, a kernel-based reinforcement learning algorithm is employed to determine the hyperparameters of the chosen potential field; (ii) guaranteeing that joint limits are respected when adjusting trajectories in the process of avoiding obstacles. During obstacle avoidance, joint trajectories are usually modified according to the effects of the potential field. Therefore, the evolution of joint trajectories shall be constrained by bounding them within the allowable range; (iii) determining proper control commands for robots such that potential human-robot interaction is safe. To do so, We propose to control the robot with a minimal intervention controller rooted in linear quadratic tracking.

The rest of the paper is organized as follows: section 2 reviews the previous work related to our problem. Section 3 presents the proposed framework for learning to avoid obstacles with minimal intervention control. Subsequently, section 4 reports the results of the toy example as well as the experiments on the iCub humanoid robot to show the effectiveness of the proposed method. Finally, conclusions and future routes of research are given in section 5.

## 2. Related Work

In general, obstacle avoidance is a classical topic. Due to its great significance, it has been extensively studied in a broad range of fields not only limited to robotics but also computer graphics, computer aided design, urban planning, etc. Among the considerable works dedicated to the topic, obstacle avoidance can be roughly classified into two categories: motion planning and reactive methods.

Sampling-based motion planning algorithms normally rely on planners, such as Probabilistic Roadmap (PRM) (Kavraki et al., 1996) and Rapidly-exploring Random Tree (RRT) algorithms (LaValle, 1998), along with their numerous extensions. In order to facilitate collision detection of the samples, polyhedrons are usually used as proxies for robots and obstacles. Collision avoidance strategies elicited from sampling-based motion planning usually could generate globally optimal trajectories, but become computationally expensive and time consuming in the case of high-dimensional multi-body problems or narrow-passage problems (Ruan et al., 2018). Optimization-based techniques can also be employed for obstacle avoidance. The collision-free trajectory can be obtained by optimizing the cost function formulated by a combination of obstacle cost and other indexes, such as smoothness. Various strategies for optimization could be applied to motion planning as well. For example, Zucker et al. (2013) presented a trajectory optimization procedure based on covariant descent. However, considering that gradient-based methods could get stuck in local optima, Kalakrishnan et al. (2011) proposed to instead use a derivative-free stochastic optimization approach to motion planning. The optimized trajectory is obtained by rolling out a series of noisy trajectories and the candidate solution is updated with the received cost with no gradient information required in the process. Also, in order to speed up the optimization process, Schulman et al. (2014) proposed to find collision-free trajectories using sequential convex optimization where collision is penalized with a hinge loss.

By contrast, reactive methods can make sure that robots can behave in response to the sensed obstacles in real time. Yet, the limitations lie in the design of priority assignment between the ongoing tasks as well as the obstacle avoidance task. Therefore, the solution is usually satisfying local conditions and thus suboptimal. In addition, there are also stability issues regarding reactive methods, as identified by Koren and Borenstein (1991).

In the framework of PbD, obstacle avoidance is usually realized with the help of potential fields, i.e., collision-free movement is generated by a repellent force obtained from a gradient of a potential field centered around the obstacle (Khatib, 1986). Kim and Khosla (1992) proposed a new formulation of the artificial potential field for obstacle avoidance using harmonic functions. A clear advantage of harmonic functions is that it can completely eliminate local minima in a cluttered environment. In the spirit of harmonic potential functions, within the context of dynamical-system-based robot control, Khansari-Zadeh and Billard (2012) proposed a real-time obstacle avoidance approach that can steer robot motions generated by the dynamical system. Since such modification happens locally, the dynamical system (DS) can still be kept globally stable, i.e., all trajectories can reach the target point. In addition, the method can handle multiple obstacles without changing the equilibrium of the original dynamics. Huber et al. (2019) further extended such method to the case of moving convex and star-shaped concave obstacles. The impenetrability of the obstacles' hull and asymptotic stability at a final goal location was proven by contraction theory.

**Figure 1:**
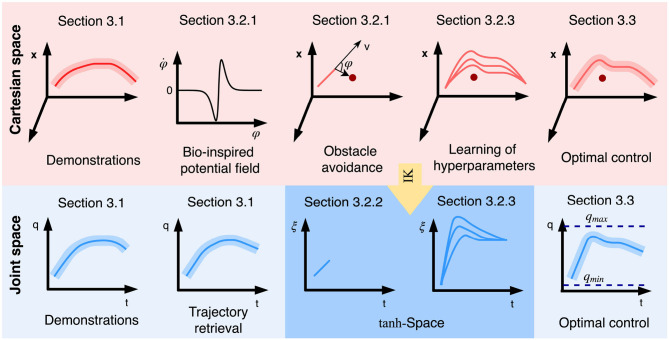
Illustration of the proposed approach to obstacle avoidance within hybrid imitation learning. First, multiple demonstrations are presented to the robot and the statistical information are encoded by GMR. Subsequently, with the help of Gaussian product between Cartesian trajectory and joint trajectory, the inconsistency from both spaces are unified. A bio-inspired potential field is employed for obstacle avoidance, since it can better reserve the fidelity against the original trajectory. The hyperparameters of the potential field are determined by reinforcement learning with the learned trajectory driven by a minimal intervention controller.

**Figure 2:**
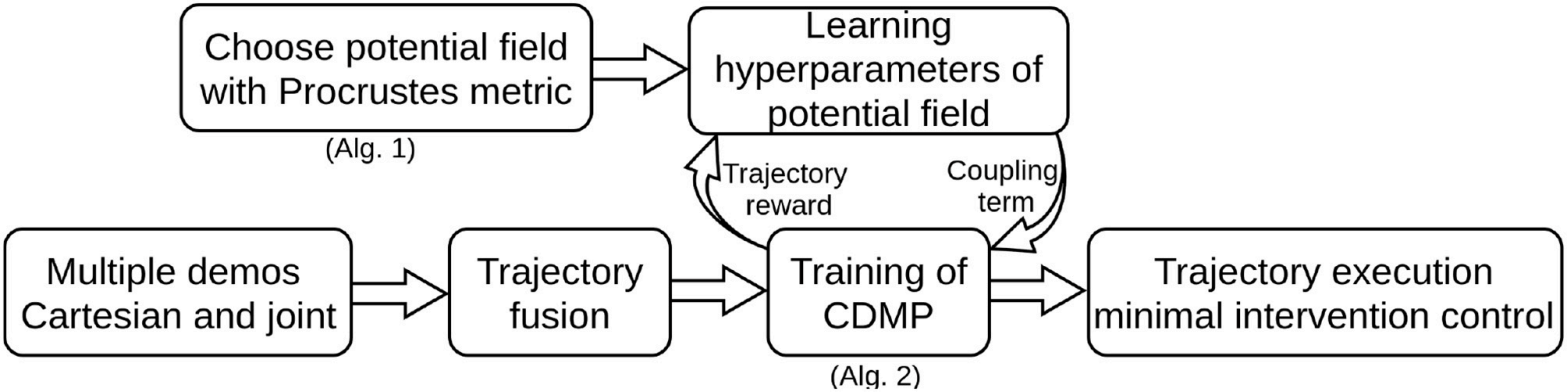
Flowchart for illustration of the proposed algorithmic framework.

Compared with previous work on obstacle avoidance within PbD, the contribution of our proposed method focuses on tackling the aforementioned three subproblems. To address problem (i), a novel imitation metric is provided such that the performance of different potential fields can be quantified. Based on such imitation metric, we can evaluate similarity between the trajectories modified due to obstacle avoidance as well as the original demonstrated trajectories. The hyperparameters of the chosen potential field are determined by a reinforcement learning algorithm. To solve problem (ii), we parameterize the joint space into exogenous states using the hyperbolic tangent function. We show that the proposed method can guarantee that the evolution of joint trajectories is always bounded within the specified range. As for problem (iii), we employ a minimal intervention control strategy based on linear quadratic tracking. The illustration of the proposed method is shown in Figure 1. In addition, a flowchart to summarize the whole procedure is shown in Figure 2.

## 3. Proposed Approach

In this section we propose an obstacle avoidance approach within PbD that aims to preserve the demonstrated trajectories. Our obstacle avoidance strategy is devised based on the principle of artificial potential field. With the goal of preserving the demonstrated trajectories, we propose to use a bio-inspired potential field called Fajen potential field proposed by Fajen and Warren (2003). The Fajen potential field is built upon the empirical evidence of how humans steer their motion for obstacle avoidance. The hyperparameters of the chosen potential field are determined by a reinforcement learning algorithm. Although the Fajen field has been used in previous works (Hoffmann et al., 2009; Rai et al., 2014), its merit over others was not elaborated explicitly. To this end, we contribute quantitative evidence to benchmark different types of potential fields and show that the Fajen potential field indeed outperforms others. Furthermore, we propose to rely on a novel imitation metric rather than RMSE to evaluate the imitation fidelity. The suggested imitation metric is based on the technique of curve similarity analysis (Dryden, 2014). Moreover, given that trajectories will be modified by the potential field unpredictably during obstacle avoidance, there are possibilities that joint trajectory evolution could exceed the allowable range. To address this issue, we employ Constrained Dynamic Movement Primitives (CDMPs), recently developed by Duan et al. (2018) so as to ensure joint trajectories are always bounded within the specified range.

### 3.1. Trajectory Retrieval From Multiple Demonstrations

In order to endow robots with variable impedance skills, a minimal intervention controller is usually used for tracking the reference trajectories. Multiple demonstrations to the robot are required to capture the statistical information underlying the reference trajectories. Assume that both Cartesian trajectory $\tau_{t}^{x}={\left[{\text{x}}_{t}^{⊤}{\overset{∙}{\text{x}}}_{t}^{⊤}\right]}^{⊤}$ and joint trajectory $\tau_{t}^{j}={\left[{\text{q}}_{t}^{⊤}{\overset{∙}{\text{q}}}_{t}^{⊤}\right]}^{⊤}$ as well as their corresponding time index *t* are recorded. For *M* demonstrations, each one having length *T*, we denote the collected dataset as ${\left\{{\left\{t_{n,m},\tau_{t,m}^{x},\tau_{t,m}^{j}\right\}}_{t=1}^{T}\right\}}_{m=1}^{M}$. In order to extract the probabilistic properties from the multiple teachings, several techniques can be employed, such as Gaussian Mixture Models (GMM) or Hidden Markov Models (HMM) (Calinon and Lee, 2016). As an example, here we employ GMM with *H* components to encode the raw training data as GMM is one of the most mature probabilistic approaches for modeling multiple demonstrations. Without loss of generality, we use ***τ*** to denote either ***τ***^*x*^ or ***τ***^*j*^. GMM first estimates the joint probability distribution with priors π_*h*_, *h* = 1, …, *H* satisfying $\overset{H}{\underset{h=1}{\sum }}\pi_{h}=1$:

(1) $$\begin{array}{r} p\left(t,\tau \right)=\overset{H}{\underset{h=1}{\sum }}\pi_{h}N\left(\mu_{h},\Sigma_{h}\right), \end{array}$$

where

(2) $$\begin{array}{r} \mu_{h}=\left[\begin{array}{c} \mu_{t,h}\\ \mu_{\tau ,h} \end{array}\right], \end{array}$$

(3) $$\begin{array}{r} \Sigma_{h}=\left[\begin{array}{cc} \Sigma_{tt,h} & \Sigma_{t\tau ,h}\\ \Sigma_{\tau t,h} & \Sigma_{\tau \tau ,h} \end{array}\right]. \end{array}$$

Furthermore, Gaussian Mixture Regression (GMR) is employed to retrieve the probabilistic trajectory (Calinon and Lee, 2016). The corresponding output with respect to a query point *t* is formulated by a conditional probability distribution:

(4) $$\begin{array}{r} \hat{\tau }\left(t\right)\sim \overset{H}{\underset{h=1}{\sum }}w_{h}\left(t\right)N({\hat{\mu }}_{h}\left(t\right),{\hat{\Sigma }}_{h}\left(t\right)), \end{array}$$

where *w*_*h*_(*t*) are the activation functions defined as

(5) $$\begin{array}{r} w_{h}\left(t\right)=\frac{\pi_{h}N\left(t|\mu_{t,h},\Sigma_{tt,h}\right)}{\sum_{i=1}^{H}\pi_{i}N\left(t|\mu_{t,i},\Sigma_{tt,i}\right)}, \end{array}$$

with

(6) $$\begin{array}{r} {\hat{\mu }}_{h}\left(t\right)=\mu_{\tau ,h}+\Sigma_{\tau t,h}\Sigma_{tt,h}^{-1}\left(t-\mu_{t,h}\right), \end{array}$$

(7) $$\begin{array}{r} {\hat{\Sigma }}_{h}\left(t\right)=\Sigma_{\tau \tau ,h}-\Sigma_{\tau t,h}\Sigma_{tt,h}^{-1}\Sigma_{t\tau ,h}. \end{array}$$

Note that (4) is usually approximated by a unimodal output distribution for robot control. By resorting to the law of total mean and variance, the approximated normal distribution $N\left({\hat{\mu }}_{t}^{u},{\hat{\Sigma }}_{t}^{u}\right)$ can be derived as

(8) $$\begin{array}{l} \begin{array}{l} {\hat{\mu }}_{t}^{u}=\overset{H}{\underset{h=1}{\sum }}w_{h}\left(t\right){\hat{\mu }}_{h}\left(t\right),\\ {\hat{\Sigma }}_{t}^{u}=\overset{H}{\underset{h=1}{\sum }}w_{h}\left(t\right)({\hat{\Sigma }}_{h}\left(t\right)+{\hat{\mu }}_{h}\left(t\right){\hat{\mu }}_{h}{\left(t\right)}^{⊤})-{\hat{\mu }}_{t}^{u}{\hat{\mu }}_{t}^{u⊤}. \end{array} \end{array}$$

After applying GMR, we obtain ${\hat{\tau }}^{x}\sim N\left({\hat{\mu }}^{\tau x},{\hat{\Sigma }}^{\tau x}\right)$ for Cartesian trajectories where ${\hat{\mu }}^{\tau x}={\left[{\hat{\mu }}^{x⊤}{\hat{\overset{∙}{\mu }}}^{x⊤}\right]}^{⊤}$, and ${\hat{\tau }}^{j}\sim N\left({\hat{\mu }}^{\tau j},{\hat{\Sigma }}^{\tau j}\right)$ for joint trajectories where ${\hat{\mu }}^{\tau j}={\left[{\hat{\mu }}^{j⊤}{\hat{\overset{∙}{\mu }}}^{j⊤}\right]}^{⊤}$. One issue arising here is that there is inconsistency emerging between Cartesian and joint constraints due to multiple demonstrations. Such phenomena are usually referred to as *competing constraints* in the literature (Calinon and Billard, 2008). In order to unify the constraints from both spaces, we employ the Gaussian product for the fusion of Cartesian and joint trajectories as in Calinon and Billard (2008). To obtain the corresponding joint trajectory **q** that satisfies the corresponding Cartesian constraints **x**, a Jacobian-based inverse kinematics technique is employed:

(9) $$\begin{array}{l} \begin{array}{l} {\text{q}}_{t}^{x}={\text{q}}_{t-1}^{x}+{\text{J}}^{†}\left({\text{x}}_{t}-{\text{x}}_{t-1}\right)+{\text{J}}^{N}\left({\text{q}}_{t}-{\text{q}}_{t-1}\right),\\ {\overset{∙}{\text{q}}}_{t}^{x}={\text{J}}^{†}{\overset{∙}{\text{x}}}_{t}+{\text{J}}^{N}{\overset{∙}{\text{q}}}_{t}, \end{array} \end{array}$$

where **J**^†^ = **J**^⊤^(**JJ**^⊤^)^−1^ denotes the Moore-Penrose pseudo inverse of **J** and **J**^*N*^ = **I**−**J**^†^**J** is a null-space matrix that projects the additional secondary task into the null space of robot movement. In this case, the joint trajectories ${\hat{\tau }}^{j}\sim N\left({\hat{\mu }}^{\tau j},{\hat{\Sigma }}^{\tau j}\right)$ retrieved from demonstrations are set as the secondary task and thus represent null-space movement. Upon transformation from Cartesian to joint space, the obtained probabilistic joint trajectory also satisfies Gaussian distribution ${\text{q}}^{C}\sim N\left(\mu^{C},\Sigma^{C}\right)$ with mean ***μ***^*C*^ and variance ***Σ***^*C*^ given by:

(10) $$\begin{array}{r} \begin{array}{c} \mu^{C}={\tilde{\text{J}}}^{†}{\hat{\mu }}^{\tau x}+{\tilde{\text{J}}}^{N}{\hat{\mu }}^{\tau j}+\Lambda_{t-1},\\ \Sigma^{C}={\tilde{\text{J}}}^{†}{\hat{\Sigma }}^{\tau x}{\tilde{\text{J}}}^{†⊤}+{\tilde{\text{J}}}^{N}{\hat{\Sigma }}^{\tau j}{\tilde{\text{J}}}^{N⊤}, \end{array} \end{array}$$

where $\Lambda_{t-1}={\left[{\left[\mu_{t-1}^{C}-J^{†}{\hat{\mu }}_{t-1}^{x}-J^{N}{\hat{\mu }}_{t-1}^{j}\right]}^{⊤}0^{⊤}\right]}^{⊤}$, ${\tilde{\text{J}}}^{†}=diagblock\left({\text{J}}^{†},{\text{J}}^{†}\right)$ and ${\tilde{\text{J}}}^{N}=diagblock\left({\text{J}}^{N},{\text{J}}^{N}\right)$. To address the competing constraints, the final probabilistic trajectory $\hat{\text{q}}\sim N\left(\hat{\mu },\hat{\Sigma }\right)$ is retrieved by fusion of Cartesian and joint constraints with the help of the Gaussian product (Calinon and Billard, 2008). That is, $\hat{\text{q}}\sim N\left(\hat{\mu },\hat{\Sigma }\right)\propto N\left(\mu^{C},\Sigma^{C}\right)\times N\left({\hat{\mu }}^{\tau j},{\hat{\Sigma }}^{\tau j}\right)$ with mean $\hat{\mu }$ and variance $\hat{\Sigma }$ given by Rasmussen (2003):

(11) $$\begin{array}{l} \begin{array}{l} \hat{\mu }=\bar{\Sigma }({\left(\Sigma^{C}\right)}^{-1}\mu^{C}+{\left({\hat{\Sigma }}^{\tau j}\right)}^{-1}{\hat{\mu }}^{\tau j}),\\ \hat{\Sigma }=({\left(\Sigma^{C}\right)}^{-1}+{\left({\hat{\Sigma }}^{\tau j}\right)}^{-1}{)}^{-1}. \end{array} \end{array}$$

By executing the retrieved probabilistic trajectory, robots are able to satisfy both Cartesian and joint constraints simultaneously.

### 3.2. Learning to Avoid Obstacles

Once the probabilistic trajectories are retrieved, the robot will be required to track the reference trajectories in presence of obstacles. We present a novel imitation metric other than the simple sum of squares in section 3.2.1 in order to compare the performance of different potential fields. The joint limit avoidance issue is addressed in section 3.2.2. Subsequently, section 3.2.3 formulates the search of the optimal potential field hyperparameters in terms of a Reinforcement Learning problem.

#### 3.2.1. Imitation Metric for Potential Field

Since we expect robots to behave like humans during the process of obstacle avoidance, our choice for the potential field is the Fajen potential field, which is derived from a bio-inspired perspective (Fajen and Warren, 2003). The basic idea behind Fajen field is to first calculate the angle between the current velocity and the direction toward the obstacle. Given this angle, the method determines how much to change the steering direction in order to keep away from the obstacle. The steering effect from the Fajen potential field is used to design the coupling term:

(12) $$\begin{array}{r} \text{p}\left(\text{x}\right)=\gamma \text{R}\overset{∙}{\text{x}}\varphi e^{-\beta \varphi }, \end{array}$$

with

(13) $$\begin{array}{r} \varphi =\arccos\left(\frac{{\left(\text{o}-\text{x}\right)}^{⊤}\dot{\text{x}}}{\left|\text{o}-\text{x}||\dot{\text{x}}\right|}\right), \end{array}$$

(14) $$\begin{array}{r} \text{r}=\left(\text{o}-\text{x}\right)\times \overset{∙}{\text{x}}, \end{array}$$

where **R** is a rotation matrix with axis **r** and rotation angle π/2, **o** denotes the position of the obstacle in Cartesian space and φ is the angle between the velocity of the end-effector $\overset{∙}{\text{x}}$ and the vector **o**−**x**, which is always positive.

As there are a number of other potential fields that can also be used for obstacle avoidance within PbD, an interesting issue arising is how to compare the performance of different potential fields. In order to evaluate the reproduction quality of the trajectory, imitation metrics are required. Traditionally, imitation metrics are mainly formulated as a weighted sum of squares of differences between the reproduced and the demonstrated trajectories, where the weights usually come from the variance matrix across multiple demonstrations (Billard et al., 2008; Calinon and Lee, 2016; Huang et al., 2018). Such evaluation metric is improper in the case of obstacle avoidance since the trajectory shapes are changed much more severely with respect to straightforward reproduction.

Here, we employ a novel perspective on the formalism of imitation metrics, such that the effects of different potential fields can be fairly evaluated. Specifically, we propose to formulate the imitation similarity metric from the perspective of curve similarity analysis (Dryden, 2014). In general, the technique of curve similarity analysis has a wide rage of applications, such as signal alignment, DNA matching, signature comparison, etc. and is very pertinent to our situation (Mitchel et al., 2018). As there are a number of curve similarity analysis methods, the Procrustes distance is used for our case as an example (Dryden, 2014).

Overall, the Procrustes distance facilitates shape analysis by removing relative translational, scaling, and rotational components. Here we consider how to calculate the Procrustes distance *d*(*X*^1^, *X*^2^) between two trajectories $X^{1}={\left\{{\left[x_{k}^{1},y_{k}^{1},z_{k}^{1}\right]}^{⊤}\right\}}_{k=1}^{K}$ and $X^{2}={\left\{{\left[x_{k}^{2},y_{k}^{2},z_{k}^{2}\right]}^{⊤}\right\}}_{k=1}^{K}$ with *K* the length. First, the translational component will be removed. To this end, all the trajectory points are translated as a whole such that the mean value of all the points coincide with the origin point. The mean value ${\bar{X}}^{i}$ of all the points for trajectory *i* is calculated as

(15) $$\begin{array}{r} {\bar{X}}^{i}=\frac{\sum_{k}X_{k}^{i}}{K}. \end{array}$$

Similarly, the scaling component is removed by normalizing the root mean square distance between the points and the origin point. The scaling factor *s*^*i*^ of trajectory *i* is calculated as

(16) $$\begin{array}{r} s^{i}=\sqrt{\frac{\sum_{k}{\left(X_{k}^{i}-{\bar{X}}^{i}\right)}^{2}}{K}}. \end{array}$$

The scale of trajectory *i* is normalized by

(17) $$\begin{array}{r} {\tilde{X}}_{k}^{i}=\frac{X_{k}^{i}-{\bar{X}}^{i}}{s^{i}} \end{array}$$

The removal of the rotation effect is not straightforward because the calculation of the optimal rotation matrix **W** requires to solve an optimization problem

(18) $$\begin{array}{r} \text{W}=\underset{\Upsilon }{\arg\max}\underset{k}{\sum }\|\Upsilon {\tilde{X}}_{k}^{1}-{\tilde{X}}_{k}^{2}{\|}^{2}, \end{array}$$

where ||·|| denotes the Euclidean norm of a vector. It can be verified that the optimal rotation matrix is given by Dryden (2014)

(19) $$\begin{array}{r} \text{W}=\text{U}\Sigma^{\prime }{\text{V}}^{⊤}, \end{array}$$

where **U*Σ*V**^⊤^ is the Singular Value Decomposition (SVD) of the matrix $\underset{k}{\sum }{\tilde{X}}_{k}^{2}{\tilde{X}}_{k}^{1⊤}$. In order to make **W** a valid rotation matrix, i.e., det(**W**)>0 where det(·) denotes the determinant of a matrix, ***Σ*** is modified into ***Σ***′ by replacing its smallest singular value with the sign of det(**UV**^⊤^), and the other singular values with 1. Finally, the Procrustes distance is given by

(20) $$\begin{array}{r} d\left(X^{1},X^{2}\right)=\sqrt{\underset{k}{\sum }\|{\tilde{X}}_{k}^{1}-\text{W}{\tilde{X}}_{k}^{2}{\|}^{2}}. \end{array}$$

The process to calculate the Procrustes between two trajectories is summarized in Algorithm 1.

**Algorithm 1:** Procrustes distance between trajectories

**Input:** Trajectories *X*^1^ and *X*^2^
**Output:** Procrustes distance *d*(*X*^1^, *X*^2^)
1: Compute ${\bar{X}}^{i}$ as per (15)
2: Compute *s*^*i*^ as per (16)
3: **for** *k* = 1 to *K* **do**
4: $X_{k}^{i}\leftarrow \left(X_{k}^{i}-{\bar{X}}^{i}\right)/s^{i}$
5: **end for**
6: $\text{U}\Sigma {\text{V}}^{⊤}\leftarrow \text{SVD}\left(\underset{k}{\sum }X_{k}^{2}X_{k}^{1⊤}\right)$
7: **if** det(**UV**) <0 **then**
8: min(***Σ***) ← sign(det(**UV**))
9: nonmin(***Σ***) ← 1
10: **end if**
11: Compute *d* as per (20)
12: **return** *d*

#### 3.2.2. Joint Limit Avoidance

Once a suitable type of potential field has been chosen, we are ready to modify robot trajectories for obstacle avoidance. One problem here is how to guarantee that joint limits are respected. It can be conceived that the modified trajectories are very susceptible to the strength of the potential field and a strong field could drive joint trajectories out of the allowable range. To cope with such issue, we will drive the robot's trajectories using our recently developed Constrained Dynamic Movement Primitives (CDMPs) (Duan et al., 2018). CDMPs are derived by parameterizing the original trajectory using the hyperbolic tangent function $\tanh\left(x\right)=\frac{\exp\left(x\right)-\exp\left(-x\right)}{\exp\left(x\right)+\exp\left(-x\right)}$. We make modifications on the hyperbolic tangent space such that the joint trajectories will always evolve within the given bound. Formally, assume the joint limits are determined by **q**_min_ and **q**_max_. The feasible joint space in terms of the exogenous variable ***ξ*** is as follows

(21) $$\begin{array}{r} \text{q}\left(\xi \right)={\text{q}}_{\text{e}}\tanh\left(\xi \right)+{\text{q}}_{\text{o}}, \end{array}$$

where ${\text{q}}_{\text{e}}=\text{diag}(\frac{1}{2}\left({\text{q}}_{\text{max}}-{\text{q}}_{\text{min}}\right))$ and ${\text{q}}_{\text{o}}=\frac{1}{2}\left({\text{q}}_{\text{max}}+{\text{q}}_{\text{min}}\right)$. Therefore, given the desired reference trajectory **q**_d_, its transformation into tanh-space is given as

(22) $$\begin{array}{r} \xi_{\text{d}}=\text{arctanh}({\text{q}}_{\text{e}}^{-1}\left({\text{q}}_{\text{d}}-{\text{q}}_{\text{o}}\right)). \end{array}$$

**Figure 3:**
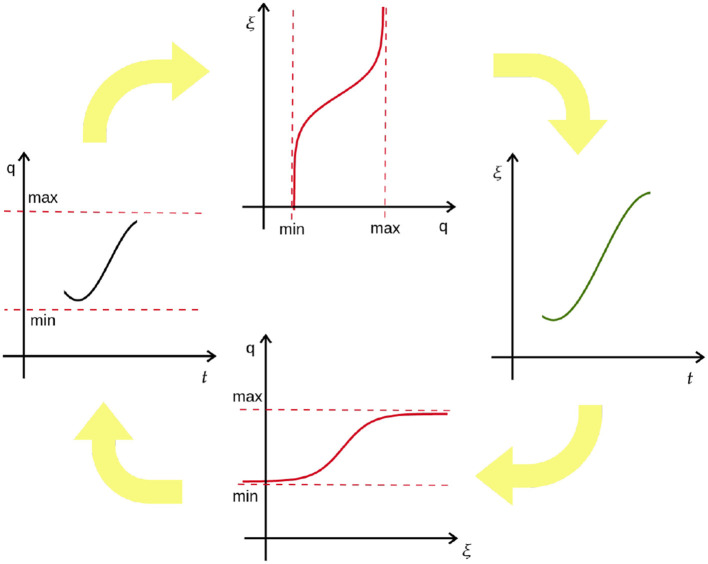
Illustration of the principle of CDMPs. Joint trajectory *q* is first transformed into the exogenous state ξ using the arctangent hyperbolic function and then transformed back using the hyperbolic tangent function. Trajectory modifications are done in *tanh*-space.

Consequently, Dynamic Movement Primitives (DMPs) are trained in tanh-space with modifications from potential field exerted therein. DMPs are described by

(23) $$\begin{array}{l} \begin{array}{l} \tau \overset{∙}{\omega }=-\alpha \omega ,\\ \tau^{2}\ddot{\xi }={\text{K}}^{p}\left(\text{g}-\xi \right)-\tau {\text{K}}^{v}\overset{∙}{\xi }+\omega \text{f}\left(\omega \right)+\text{p},\\ f_{k}\left(\omega \right)=\frac{\sum_{i=1}^{N}\phi_{i}\left(\omega \right)\chi_{k,i}}{\sum_{i=1}^{N}\phi_{i}\left(\omega \right)}, \end{array} \end{array}$$

where τ>0 denotes movement duration, α>0 is a scalar, ω is the phase variable on which the forcing term **f** depends, $\phi_{i}\left(\omega \right)={\text{e}}^{-h_{i}{\left(\omega -l_{i}\right)}^{2}}$ is the basis function with *h*_*i*_>0 and *l*_*i*_∈[0, 1] and χ_*k, i*_ is the corresponding weight. Moreover, **p** is the coupling term that modifies the DMPs trajectory according to the potential field. It should be noted that the training of DMP is the same as the usual procedure, i.e., fitting the acceleration profile with Gaussian radial basis functions. Here the only difference is that it happens in tanh-space. The working flow of CDMPs is illustrated in Figure 3 and the corresponding algorithm is summarized in Algorithm 2.

**Algorithm 2:** CDMPs

**Input:** Feasible trajectories $\hat{\text{q}}$
**Output:** Bounded trajectories $\tilde{\text{q}}$ *Initialization* : **q**_min_, **q**_max_, DMPs hyperparameters
1: Transform $\hat{\text{q}}$ into tanh-space as per (22)
2: Train DMPs as per (23)
3: Add coupling term for obstacle avoidance as per (12)
4: Transform back for $\tilde{\text{q}}$ as per (21)
5: **return** $\tilde{\text{q}}$

#### 3.2.3. Reinforcement Learning of Hyperparameters

Normally, the strength of a potential field determined by the hyperparameters can affect the performance of obstacle avoidance greatly. If the strength is too high, then the robot will react more than enough to avoid the obstacle. Yet, although joint limit cannot be exceeded thanks to CDMPs, the similarity with respect to the demonstrated behavior will be very much sacrificed. On the other hand, if the strength is too low, the robot would be in danger of hitting the obstacle. In order to find the optimal hyperparameter values, a non-parametric reinforcement learning algorithm called Cost-regularized Kernel Regression (CrKR) is employed (Kober et al., 2012). CrKR is used to learn the optimal policy that maps the state, which is the position of the obstacle, to the optimal action, which is associated with the corresponding hyperparameters. At the beginning of each trial, for a query obstacle position **s**_*o*_, the hyperparameters are sampled from the Gaussian distribution $\gamma_{i}\sim N(\gamma_{i}\left({\text{s}}_{o}\right),\sigma^{2}\left({\text{s}}_{o}\right))$ with the mean given by

(24) $$\begin{array}{r} \gamma_{i}\left({\text{s}}_{o}\right)=\text{k}{\left({\text{s}}_{o}\right)}^{⊤}{\left(\text{K}+\lambda \text{C}\right)}^{-1}\Gamma_{i}, \end{array}$$

and the variance to facilitate exploration given by

(25) $$\begin{array}{r} \sigma^{2}\left({\text{s}}_{o}\right)=k\left({\text{s}}_{o},{\text{s}}_{o}\right)+\lambda -\text{k}{\left({\text{s}}_{o}\right)}^{⊤}{\left(\text{K}+\lambda \text{C}\right)}^{-1}\text{k}\left({\text{s}}_{o}\right), \end{array}$$

where *k*(·, ·) is a kernel function; $\text{k}\left({\text{s}}_{o}\right)=\phi {\left({\text{s}}_{o}\right)}^{⊤}\Phi^{⊤}$ with ***ϕ***(·) being basis functions and ***Φ***_*i*_ = ***ϕ***(**s**_*i*_), **K** = ***ΦΦ***^⊤^, **C** = diag(*c*_1_, *c*_2_, …, *c*_*n*_) is a cost matrix, λ is a ridge factor and ***Γ***_*i*_ is a vector containing the training examples for the hyperparameters. It should be noted that the cost element *c*_*i*_ stored in the cost matrix **C** is calculated by the Procrustes distance between the collision-free trajectory ${\tilde{\tau }}_{i}$ and the demonstrated trajectory $\hat{\tau }$, i.e., $c_{i}=d\left({\tilde{\tau }}_{i},\hat{\tau }\right)$. The learning process terminates when the distance between the robot end-effector and the obstacle is smaller than the threshold.

### 3.3. Minimal Intervention Control

To track the reference trajectory $\tilde{\mu }$ with optimal control inputs ${\text{U}}^{*}={\left[{\text{u}}_{t}^{⊤}{\text{u}}_{t+1}^{⊤}\ldots {\text{u}}_{t+m-1}^{⊤}\right]}^{⊤}$, a minimal intervention control strategy can be employed (Calinon et al., 2014). To start with, an optimization problem is formulated as

(26) $$\begin{array}{r} {\text{U}}^{*}=\arg\underset{\text{U}\in {\mathbb{R}}^{m}}{\min}\overset{t+m}{\underset{i=t}{\sum }}({\left(\tau_{i}^{j}-{\tilde{\mu }}_{i}\right)}^{⊤}{\hat{\Sigma }}_{i}^{-1}\left(\tau_{i}^{j}-{\tilde{\mu }}_{i}\right))+\overset{t+m-1}{\underset{i=t}{\sum }}{\text{u}}_{i}^{⊤}{\text{R}}_{i}{\text{u}}_{i}, \end{array}$$

where **R**_*i*_ is a positive definite weight matrix.To find the analytical solution U^*^, we formulate joint dynamics in terms of a double integrator:

(27) $$\begin{array}{r} \underset{\tau_{t+1}^{j}}{\underset{︸}{\left[\begin{array}{c} {\text{q}}_{t+1}\\ {\overset{∙}{\text{q}}}_{t+1} \end{array}\right]}}=\underset{\text{A}}{\underset{︸}{\left[\begin{array}{cc} \text{I} & \text{I}\Delta t\\ 0 & \text{I} \end{array}\right]}}\underset{\tau_{t}^{j}}{\underset{︸}{\left[\begin{array}{c} {\text{q}}_{t}\\ {\overset{∙}{\text{q}}}_{t} \end{array}\right]}}+\underset{\text{B}}{\underset{︸}{\left[\begin{array}{c} 0\\ \text{I}\Delta t \end{array}\right]}}{\text{u}}_{t}. \end{array}$$

By applying (27) repetitively, we have

(28) $$\begin{array}{r} \underset{{\bar{\tau }}^{j}}{\underset{︸}{\left[\begin{array}{c} \tau_{t}^{j}\\ \tau_{t+1}^{j}\\ \tau_{t+2}^{j}\\ \vdots \\ \tau_{t+m}^{j} \end{array}\right]}}=\underset{\bar{\text{A}}}{\underset{︸}{\left[\begin{array}{c} \text{I}\\ \text{A}\\ {\text{A}}^{2}\\ \vdots \\ {\text{A}}^{m} \end{array}\right]}}\tau_{t}^{j}+\underset{\bar{\text{B}}}{\underset{︸}{\left[\begin{array}{cccc} 0 & 0 & \cdots & 0\\ \text{B} & 0 & \cdots & 0\\ \text{A}\text{B} & \text{B} & \cdots & 0\\ \vdots & \vdots & \ddots & \vdots \\ {\text{A}}^{m-1}\text{B} & {\text{A}}^{m-2}\text{B} & \cdots & \text{B} \end{array}\right]}}\underset{\text{U}}{\underset{︸}{\left[\begin{array}{c} {\text{u}}_{t}\\ {\text{u}}_{t+1}\\ \vdots \\ {\text{u}}_{t+m-1} \end{array}\right]}}. \end{array}$$

Therefore, the original optimization problem as in (26) can be re-written compactly:

(29) $$\begin{array}{l} f\left(\text{U}\right)=({\left({\bar{\tau }}^{j}-\bar{\mu }\right)}^{⊤}{\bar{\Sigma }}^{-1}\left({\bar{\tau }}^{j}-\bar{\mu }\right))+{\text{U}}^{⊤}\bar{\text{R}}\text{U} \end{array}$$

where

(30) $$\begin{array}{l} \begin{array}{l} \bar{\mu }={\left[{\tilde{\mu }}_{t}^{⊤},{\tilde{\mu }}_{t+1}^{⊤},\ldots ,{\tilde{\mu }}_{t+m}^{⊤}\right]}^{⊤},\\ \bar{\Sigma }=\text{blockdiag}\left({\hat{\Sigma }}_{t},{\hat{\Sigma }}_{t+1},\ldots ,{\hat{\Sigma }}_{t+m}\right),\\ \bar{\text{R}}=\text{blockdiag}\left({\text{R}}_{t},{\text{R}}_{t+1},\ldots ,{\text{R}}_{t+m-1}\right). \end{array} \end{array}$$

By inserting (28) into (29) and setting the derivative with respect to **U** equal to 0, the optimal control policy U^*^ can be calculated as

(31) $$\begin{array}{l} {\text{U}}^{*}=({\bar{\text{B}}}^{⊤}{\bar{\Sigma }}^{-1}\bar{\text{B}}+\bar{\text{R}}{)}^{-1}({\bar{\text{B}}}^{⊤}{\bar{\Sigma }}^{-1}\left(\bar{\mu }-\bar{\text{A}}\tau_{t}^{j}\right)). \end{array}$$

The complete procedure for the proposed approach to obstacle avoidance with minimal intervention control is summarized in Algorithm 3.

**Algorithm 3:** Learning to avoid obstacles with minimal intervention control

1: **Trajectory retrieval**:
– Collect dataset ${\left\{{\left\{t_{n,m},\tau_{t,m}^{x},\tau_{t,m}^{j}\right\}}_{t=1}^{T}\right\}}_{m=1}^{M}$.
– Extract statistical information as per (8).
– Fuse Cartesian and joint trajectories as per (11).
2: **Obstacle avoidance**
– Choose potential field as per Algorithm 1.
– Train CDMPs as per Algorithm 2.
– Hyperparameters learning as per (24) and (25).
3: **Minimal intervention control**:
– Apply control law as per (31).

## 4. Experiments

This section illustrates the effectiveness of the proposed approach by reporting the results of two evaluative experiments. The first experiment is a toy example where the comparison between different potential fields is conducted. With the help of the toy example, the necessity of introducing a novel imitation metric under obstacle avoidance is unveiled. Subsequently, the second experiment is devised as a transportation task under obstacle avoidance, following the proposed framework to obstacle avoidance under PbD. The real experiment is conducted on an iCub, a full-body child-size humanoid robot (Natale et al., 2017).

### 4.1. Toy Example

By convention, the imitation metric between the reproduced trajectory and the demonstrated one is designed as RMSE. However, in the context of obstacle avoidance, we show in the toy example that RMSE is not a proper imitation metric as it can not reflect the real imitation fidelity. Therefore, a novel imitation metric instead of RMSE is required to measure the imitation fidelity. As discussed earlier, we choose the imitation metric by resorting to the technique of curve similarity analysis. Specifically, the Procrustes distance is employed to replace the RMSE so as to compare the performance of different potential fields. In our toy example, the performances of the Fajen potential field as provided in (12) and the Khatib potential field (Khatib, 1986) are compared against each other. The mechanism of the Khatib potential field for obstacle avoidance is that the repulsive force becomes larger as the manipulator moves closer to the obstacle:

(32) $$\begin{array}{l} U\left(\text{x}\right)=\{\begin{array}{ll} \frac{\eta }{2}{\left(\frac{1}{\rho \left(\text{x}\right)}-\frac{1}{\rho_{0}}\right)}^{2}, & \rho \left(\text{x}\right)\leq \rho_{0},\\ 0, & \rho \left(\text{x}\right)>\rho_{0}, \end{array} \end{array}$$

where ρ(**x**) is the distance between the current position and the obstacle, η is a gain term and ρ_0_ is called the threshold distance to the obstacle point. The coupling term for obstacle avoidance with Khatib potential field is calculated by deriving the gradient, i.e., *p*(**x**) = −∇*U*(**x**).

**Figure 4:**
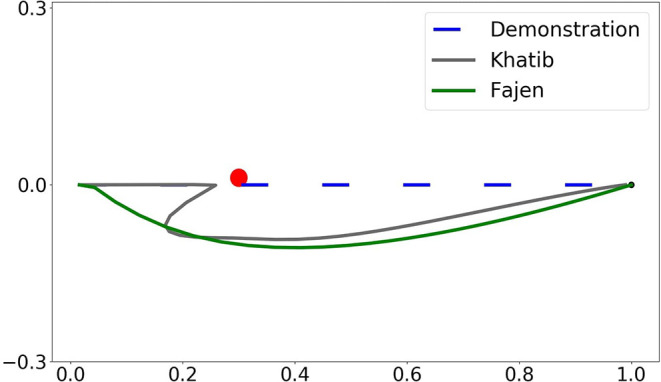
Illustration of the comparison between Khatib and Fajen potential field with Procrustes distance.

The comparison of obstacle avoidance performance between the Khatib potential field and the Fajen potential field is shown in Figure 4. A point moves from the starting point (0, 0) to the goal point (1, 0) with an obstacle located at midway (0.3, 0). The parameters used for Khatib potential field are chosen as η = 0.12 and *p*_0_ = 0.15, while the parameters used for the Fajen potential field are chosen as γ = 2000 and β = 20/π.

As can be seen in Figure 4, by employing Khatib potential field, the area enclosed by the distorted obstacle avoidance trajectory and the demonstrated one is smaller than that of Fajen field. In this imitation metric, Khatib potential field preserves higher imitation fidelity than Fajen field. However, this metric is not reasonable as one can identify intuitively that the obstacle avoidance trajectory under Fajen field should share more similarity to the demonstrated one than Khatib field. And by employing Procrustes distance for the imitation metric, indeed the obstacle avoidance trajectory under Fajen field has a smaller deviation from the demonstrated one.

Numerically, when evaluated with root mean square error, the distance is 0.37 for Khatib potential field and 0.41 for the Fajen field. However, by using the Procrustes distance as imitation metric, the distance becomes 3.06 for the Khatib field and only 0.92 for the Fajen field. In conclusion, Procrustes distance should be chosen over RMSE when the reproduction trajectory is modified irregularly. Besides, Fajen field should be preferred for obstacle avoidance as it preserves imitation fidelity better. Table 1 summarizes the quantitative comparison between two potential fields under two types of similarity metric. The code is available as Supplementary Material.

**Table 1:** Comparison of imitation metric.

	**Khatib potential field**	**Fajen potential field**
Root mean square error	0.37	0.41
Procrustes distance	3.06	0.92

### 4.2. Transportation Task

We evaluate the proposed method with a real experiment on the iCub humanoid robot. In order to show the effectiveness of the proposed framework for obstacle avoidance within PbD, a transportation task is considered as a concept-proof experiment. As iCub is a full-body humanoid robot with 56 DoFs in total, to accomplish the transportation task in our case, only the right arm having 7 DoF is activated with 3 from the shoulder, 3 from the wrist, and 1 from the elbow.

**Figure 5:**
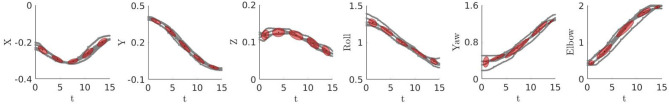
GMM modeling of demonstrated Cartesian (left three) and joint (right three) trajectories. The red ellipses represent Gaussian components and the gray trajectories denote multiple demonstrations.

**Figure 6:**
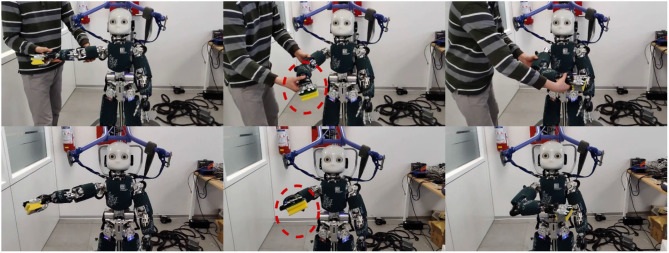
Snapshots of human demonstrations **(top row)** in a transportation task and its corresponding reproduction **(bottom row)** under an obstacle.

The conceived experimental set-up is as such: a sponge is first handed over to the robot, and then a human teacher guides the activated robot right arm to reach a final location. Finally, the robot is required to reproduce the demonstrated trajectory with the existence of an obstacle positioned midway through the demonstrated trajectory, as shown in Figure 6. During the kinesthetic teaching phase, the robot is taught the transportation task for five times. The collected dataset records both robot Cartesian and joint trajectories. In order to encode the probabilistic information underlying the multiple demonstrated trajectories, a GMM with five components is employed to model the distributions of the demonstrated Cartesian and joint trajectories, respectively. The GMM modeling results are plotted in Figure 5, and it can be observed that the trajectory segment with larger variation incurs larger covariance. The probabilistic reference trajectories to control the robot are extracted with GMR. In order to unify the inconsistency between Cartesian and joint trajectories as a result of multiple demonstrations, the trajectories from both spaces are fused by the Gaussian product as in (11). The experimental illustration is shown in Figure 6.

**Figure 7:**
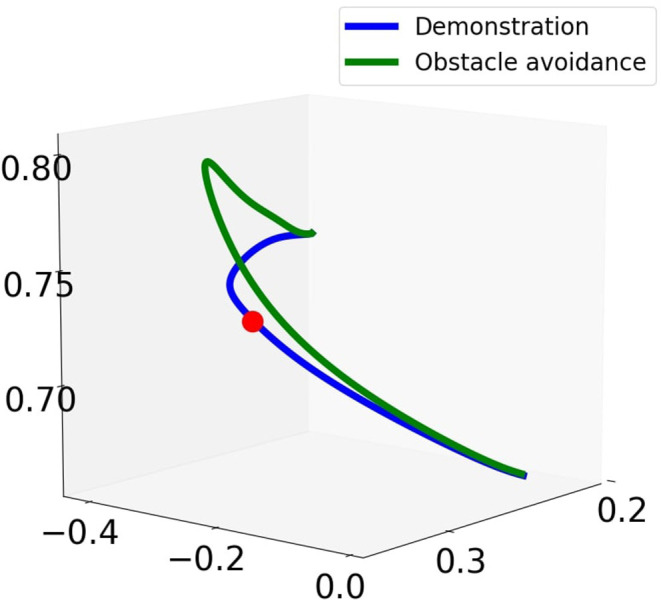
Cartesian trajectory for reproduction of demonstrated trajectory with and without obstacle.

In order to incorporate joint limit avoidance during obstacle avoidance, the retrieved trajectories are then used to train CDMPs according to Algorithm 2. For the design of the coupling term of CDMPs, the Fajen potential field is employed for obstacle avoidance. As it has been shown in the toy example, it outperforms Khatib potential field with the Procrustes distance as imitation metric. During the reproduction phase, a virtual obstacle is positioned at (0.31, −0.25, 0.73) m in Cartesian space with respect to the world frame. The snapshots of the robot reproducing the transportation tasks with the obstacle is shown in the bottom row of Figure 4. The Cartesian trajectory of the obstacle avoidance trajectory (in green) and the demonstrated trajectory (in blue) is shown in Figure 7. During the execution of the obstacle avoidance, no joint violates the corresponding joint limits.

**Figure 8:**
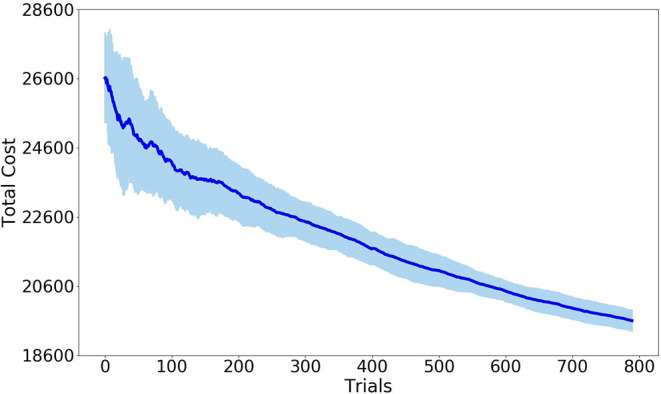
Error-bar curve of cost values with Procrustes distance as imitation metric. The solid line denotes mean values while the light blue represents the standard deviation. All quantities are expressed in meters.

The hyperparameter values of the potential field are determined by the reinforcement learning algorithm CrKR in an off-line fashion. We learn these hyperparameters as a function of the position of the obstacle. The reward used by CrKR is formulated as the Procrustes distance between the obstacle avoidance trajectory and the demonstrated one. To run CrKR, we choose the Gaussian kernel $k=\exp({\left({\text{s}}_{i}-{\text{s}}_{j}\right)}^{2})$ for the algorithm with the distance of a point from itself *k*(**s**, **s**) = 1 and a ridge factor λ = 0.5. During the preparation steps of the algorithm, the corresponding matrices **K**, **C** and **Γ** are initialized by 20 samples. The total number of trials for each run of the algorithm is 800. The cost variance is calculated by repeating the learning process for five times. The learning results are reported in Figure 8. The learning process terminates when the threshold of the minimum distance to the obstacle is triggered. When the learning process finishes, the Fajen field parameters are obtained as γ = 1260 and β = 3.2 with respect to the specified obstacle position.

Finally, the reproduction trajectory is executed with a minimal intervention controller in order to endow the robot with variable impedance skills. The cost function of the minimal intervention controller is parameterized by **R** = 10^−2^**I**. The prediction horizon is set as 10 discrete time steps.

## 5. Conclusions and Future Work

In this paper, we presented an approach to obstacle avoidance in the context of hybrid imitation learning. To exploit the probabilistic information underlying the trajectories, multiple demonstrations are taught to the robot. The initial trajectory is then retrieved from the human demonstration dataset by fusing Cartesian and joint constraints with the Gaussian product. Since there are various types of potential field, the Procrustes distance other than RMSE is employed for the benchmarking of the performance of different potential fields. As a common technique in curve similarity analysis, the Procrustes distance can better reflect the imitation fidelity between the obstacle-avoidance trajectory and the original demonstrated one. It should be noted that minimizing the Procrustean imitation metric might be more numerically expensive than square root distance. Given that the potential field would modify joint trajectories unpredictably during obstacle avoidance, joint limit avoidance is incorporated to guarantee the evolution of the modified trajectories is always bounded within the allowable range. To this end, the novel Constrained Dynamic Movement Primitives (CDMPs) method is employed. CDMPs parameterize joint trajectories with the help of the hyperbolic tangent function. By exploiting the boundedness property of the hyperbolic tangent function, the modified joint trajectories are guaranteed to evolve within the specified range. Further, in view of the fact that the performance of obstacle avoidance is quite susceptible to the hyperparameters of the potential field, a reinforcement learning algorithm is used to find the most suitable hyperparameters. The final obstacle avoidance trajectory is tracked with a minimal intervention controller to endow the robot with variable impedance capabilities.

As a preliminary attempt to address obstacle avoidance issues under PbD, a number of topics remain to be investigated for future work. For example, the position of the obstacle is given beforehand in this paper, but it could be interesting to exploit the visual system of iCub such that the obstacle position can be autonomously determined. In addition, the proposed method could be considered for extension to the case of moving obstacles or obstacles with 3D shape.

## Data Availability Statement

All datasets generated for this study are included in the article/Supplementary Material.

## Author Contributions

All authors listed have made a substantial, direct and intellectual contribution to the work, and approved it for publication.

## Conflict of Interest

The authors declare that the research was conducted in the absence of any commercial or financial relationships that could be construed as a potential conflict of interest.
